# Chest wall reconstruction with an anatomically designed 3-D printed titanium ribs and hemi-sternum implant

**DOI:** 10.1186/s41205-020-00079-0

**Published:** 2020-09-25

**Authors:** Ira Goldsmith, Peter Llewelyn Evans, Heather Goodrum, James Warbrick-Smith, Thomas Bragg

**Affiliations:** 1 Department of Cardiothoracic Surgery, Morriston Hospital, Swansea, SA6 6NL Wales, UK; 2 Maxillofacial Laboratory Services, Morriston Hospital, Swansea, SA6 6NL Wales, UK; 3 Department of Burns and Plastics Surgery, Morriston Hospital, Swansea, SA6 6NL Wales, UK

**Keywords:** Chest wall resection, Chest wall reconstruction, 3-D printed titanium implant, Chondroscarcoma

## Abstract

**Background:**

Chest wall resection following wide local excision for bone tumor results in a large defect. Reconstructing this defect is complex and requires skeletal and soft tissue reconstruction. We describe the reconstruction of a large skeletal defect with a three-dimensional (3-D) printed custom-made, anatomically designed, titanium alloy ribs and hemi-sternum implant.

**Method:**

To design the implant manual bone threshold segmentation was performed to create a 3-D virtual model of the patient’s chest and the tumor from sub-millimeter slice computed tomography (CT) scan data. We estimated the extent of resection needed to ensure tumor-free margins by growing the tumor by two cm all around.. We designed the implant using an anatomical image of the ribs and right hemi-sternum and then fabricated a 3D model of them in titanium metal using TiMG 1 powder bed fusion technology. At surgery the implant was slotted into the defect and sutured to the ribs laterally and hemi-sternum medially.

**Results:**

Histology confirmed clear all around microscopic margins. Following surgery and at 18 month follow up the patient was asymptomatic with preserved quality of life and described no pain, localized tenderness or breathlessness. There was no displacement or paradoxical movement of the implant.

**Conclusion:**

Our techniques of CT segmentation, editing, computer aided design of the implant and fabrication using laser printing of a custom-made anatomical titanium alloy chest wall ribs and hemi-sternum for reconstruction is feasible, safe and provides a satisfactory result. Hence, a patient specific 3-D printed titanium chest wall implant is another useful adjunct to the surgical approach for reconstructing large chest wall defects whilst preserving the anatomical shape, structure and function of the thorax.

## Introduction

Chondrosarcoma of the chest wall is rare, and when diagnosed requires a full thickness wide local excision of the tumor and chest wall to ensure tumor-free margins, minimize local recurrence and contribute to long-term survival [1]. However, a wide local excision of the chest wall results in a large defect and reconstructing this defect requires a combination of skeletal reconstruction and soft tissue cover [2]. The reconstruction is complex and challenging. Traditionally the skeletal reconstruction has been performed using various techniques including a mesh and methyl-methacrylate orthopedic cement prosthesis (MMCP) [3]. This can only be prepared at the time of surgery, is time consuming, difficult to mould into shape, and has the potential for dislocation resulting in paradoxical movement during respiration. The development of 3-D laser printing technology for titanium implants can help simplify this challenge and reduce the surgical operating time. We describe the use of this technology in an adult male who underwent a wide surgical excision of a chest wall chondrosarcoma followed by reconstruction of the resulting large skeletal defect with a custom-made 3-D printed anatomical ribs and hemi-sternum titanium alloy implant. The implant mimicked the anatomical contour and size of the skeletal reconstruction required.

## Methods

**Fig. 1:**
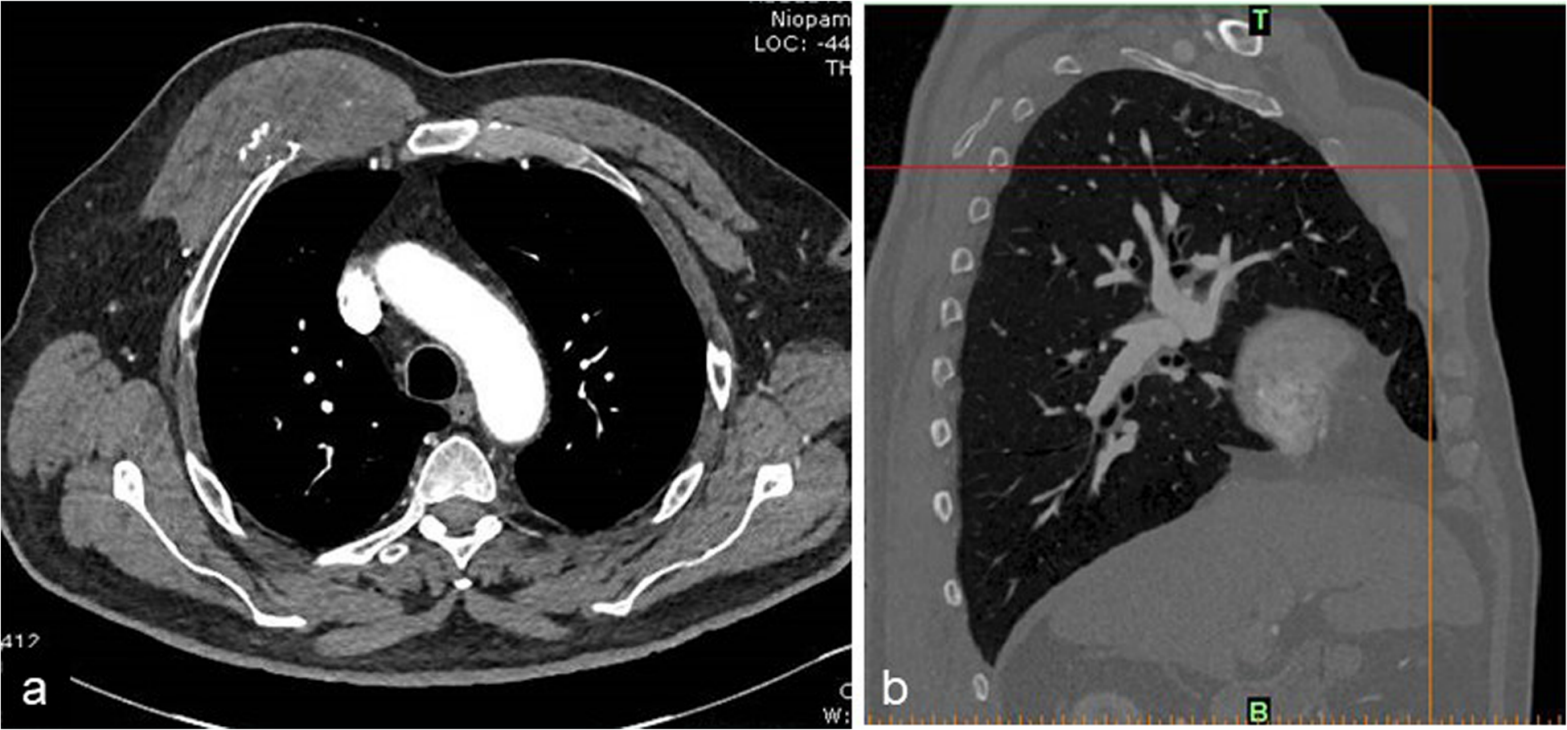
CT scan of the chest. Axial view (**a**). The tumour is located deep to the right pectoralis major muscle with scalloping of the anterior surface of the third rib. The sagittal view shows the tumour lying over the 2nd, 3rd and 4th ribs anteriorly (**b**)

A 70-year male, an ex-smoker presented to our regional cardiothoracic surgical unit at Morriston Hospital, Swansea, with a right anterior pectoral mass measuring approximately 10 cm by 9 cm estimated to have been present for about twelve months. Past medical history included type II diabetes mellitus, schizophrenia, angina with ST elevation myocardial infarction (STEMI), two episodes of transient ischemic attacks, hypothyroidism, an abdominal hernia and bilateral coarse tremors of the upper limb. He used a walking aid to walk. A chest CT scan confirmed a tumor arising in the right anterior chest wall, beneath the pectoralis major muscle and separate from the muscle but involving the underlying ribs (Fig. 1a). There was minor scalloping of the right 3rd anterior rib, and the tumor was located anterior to the 2nd, 3rd and 4th ribs (Fig. 1b). We found no radiological evidence for pulmonary, mediastinal, bony or liver lesions.. He was able to exercise using the Bruce protocol to 86% of predicted maximum heart rate without complaint of chest pain or manifesting ST-T changes or arrhythmias. Coronary angiography revealed a chronically occluded mid left anterior descending artery, which was consistent with his STEMI in 2006, and the circumflex and dominant right coronary arteries were atheromatous but unobstructed.

Histology of the tumor mass was consistent with chondrosarcoma, which for long term survival necessitated not only wide excision, but also removal of the right pectoral muscles, the second to the fourth ribs and intercostal muscles, and a portion of the right hemi-sternum [1]. Given the extent of reconstruction required, the limitations of the methyl-methacrylate method to model ribs, the need to preserve the normal thoracic anatomy and function, the importance to keep anesthesia time to a minimum, and considering the underlying coronary artery disease and co-morbidities, we considered using a custom made 3D titanium implant made in advance of surgery. Consequently, a multidisciplinary team approach was adopted to design, plan, and carry out the excision of the tumor and reconstruct the resulting defect. The MMCP option was kept as a backup alternative if a larger resection was needed at surgery. Although it would have been possible to use the techniques outlined to manufacture a processed Polymethyl methacrylate (PMMA) implant prior to surgery, the far superior biocompatibility and strength offered by a Titanium implant was deemed necessary and hence used.

### Preparation

**Fig. 2:**
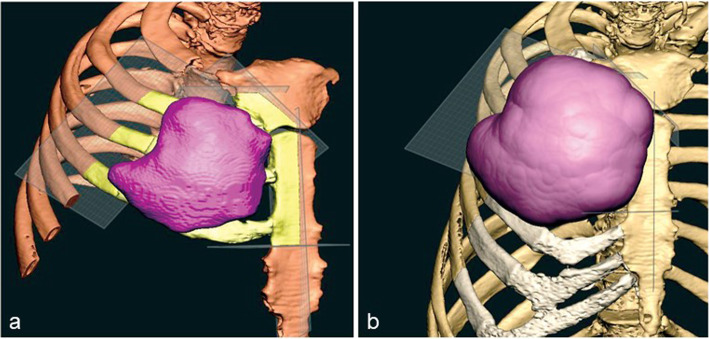
Manual bone threshold segmentation to digitally reconstruct the tumour (**a**) and enlarging the tumour digitally by 2 cm to estimate resection margins (**b**). Planes surrounding the tumour show potential resection of bone and cartilage. The area to be removed is in yellow

**Fig. 3:**
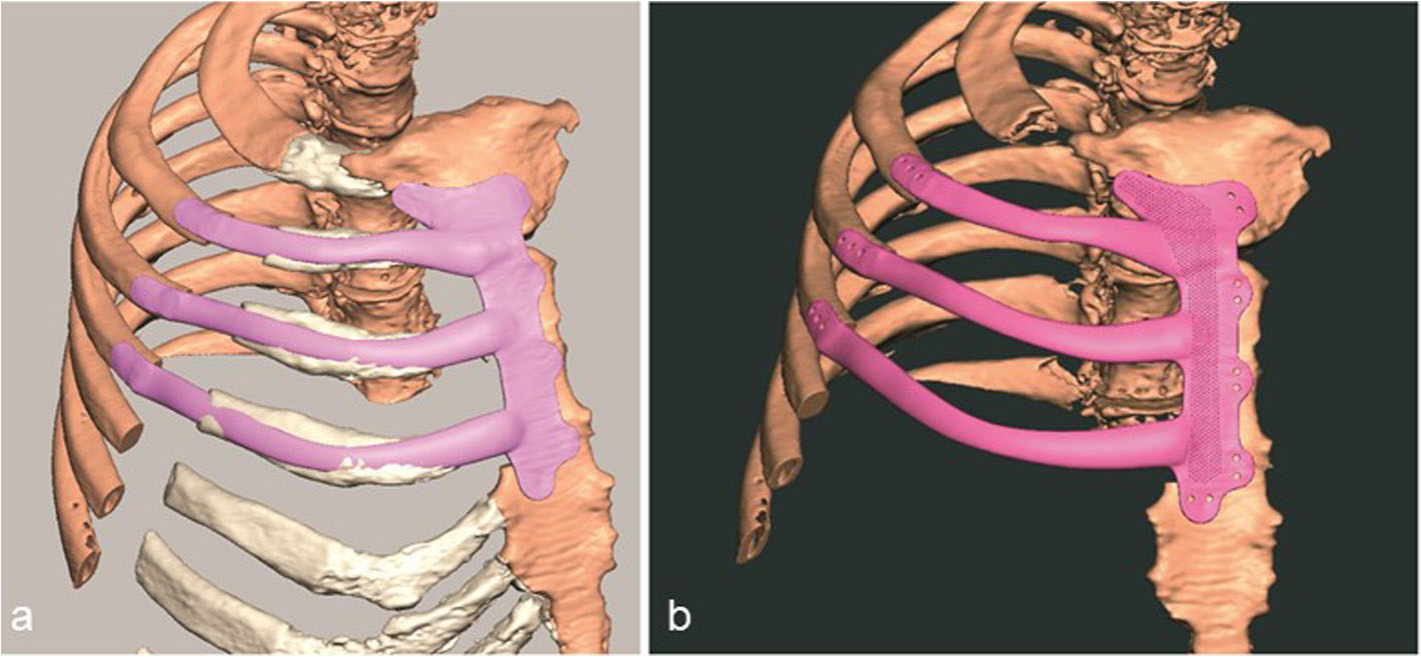
An anatomical image of the ribs and right hemi-sternum obtained digitally from the CT scan (**a**) and the titanium implant fashioned anatomically to mimic their shape and surface contours, namely the depressions and ridges of the sternum and ribs (**b**)

At our Maxillofacial Laboratory Services in Morriston Hospital, Swansea, under the guidance of the surgeons (IG), we imported sub-millimeter Digital Imaging and Communications in Medicine (DICOM) CT data into Mimics Medical 20.0 software (Materialise, Leuven, Belgium) [4, 5]. Manual bone threshold segmentation was performed to create a 3-D virtual model of the patient’s chest and tumor (Fig. 2a). This was digitally reconstructed in 3-D from the sub millimeter CT slices. As the CT scanner data varies from case to case, we used manual adjustment by eye, which has previously helped us provide best results over automatic segmentation and region growing, and was used [6]. The ribs were clearly visible on CT slices to produce the Stereolithographic (STL) files. The STL files of the ribs, the tumor and sternum were imported into Geomagic Freeform Plus software (3-D Systems, Rock Hill, United States). We estimated the extent of resection needed to ensure tumor-free margins by growing the tumor by two cm all around and achieve clear microscopic margins (Fig. 2b). An anatomical image of the ribs and right hemi-sternum to be reconstructed were then digitally obtained from the chest CT scan and the proposed titanium implant, as a single part in titanium alloy, was fashioned to mimic the shape and contours of the ribs and sternum (Fig. 3a).

**Fig. 4:**
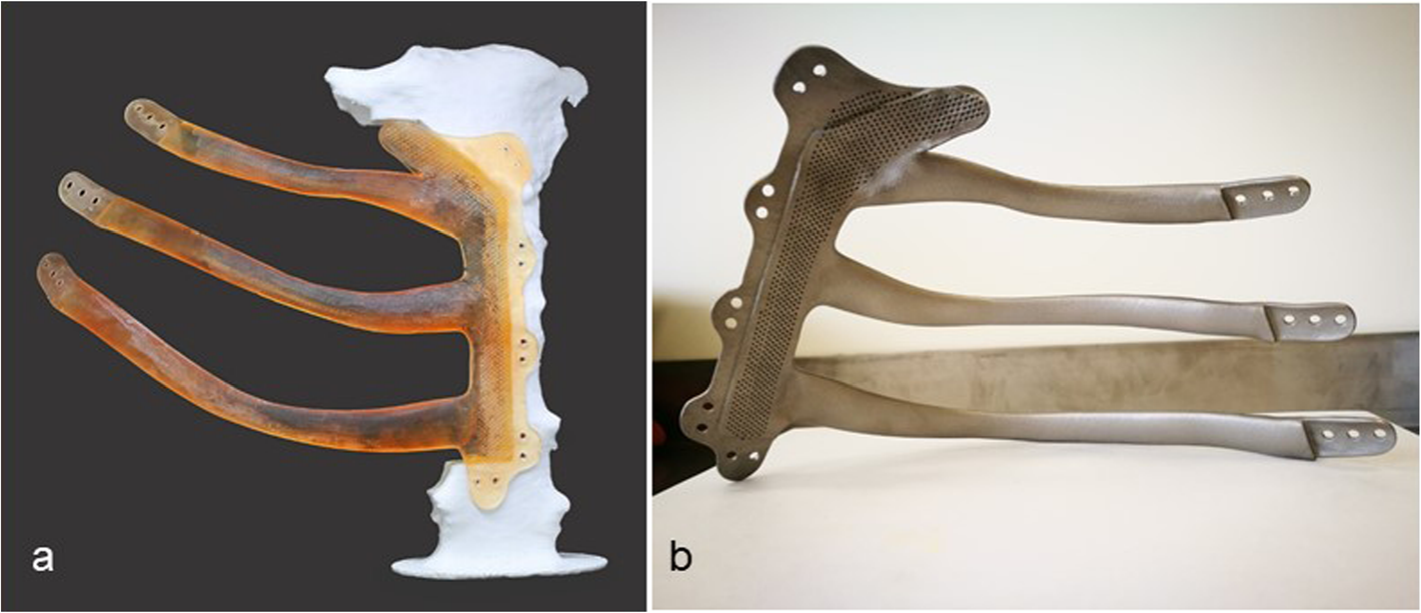
A 3-D test model of the implant tested on a 3-D printed model of the sternum showing a precise fit (**a**) and the laser printed 3-D titanium implant made by Renishaw, UK (**b**). The body of the implant was designed to be thicker than the overlapping areas to create a rabbet (step-shaped recess) for the implant to slot into the defect

At the overlapping and fixation sites, the contours of the underlying ribs laterally, and sternum medially, were carefully imitated. The aim was for the implant to anatomically fit onto the surfaces of the remaining fragments of the excised three ribs and the sternum (Fig. 3b). To keep the implant light yet robust enough to withstand the stresses placed on the chest wall, the design was limited to 1.2 mm thickness at the overlapping fixation sites [6]. The body of the implant was, however, designed to be thicker than the overlapping edges to create a rabbet (step-shaped recess) for the implant to slot into the defect and prevent any future dislocation (Fig. 4b). Suture holes were placed to attach the implant to the bone with interrupted size 5 Ethibond Excel Polyester sutures (Ethicon, Johnson and Johnson) to provide robust fixation albeit with an expectation that movement at this interface would be obligatory and hence, provide some flexibility to the rib cage during breathing (Fig. 3b).

A 3-D test model of the implant and sternum, in resin, were printed with a Formlabs Form 2 stereolithographic printer (Formlabs Form 2, Massachusetts, United States) and the proposed resection of the sternum carried out in our 3-D Maxillofacial Laboratory at Morriston Hospital in Swansea (Fig. 4a). The ribs and hemi-sternum implant was found to fit precisely the 3-D model of the defect area. Hence, the decision was made to proceed to the final implant. The implant was manufactured from Titanium alloy (Ti MG 1) by a metal powder fusion technology, selective laser melting (SLM) in (0.001 in to 0.004 in) layers carried out by Renishaw PLC at their plant in Miskin, South Wales, UK (Fig. 4b) [7]. Heat treatment was performed after printing and the implant was brought to a temperature of 850 °C, which was held for 1 h. For finishing of the Ti part, all parts underwent bead blasting with alumina 60um grit / honite, coarse flap wheel grinding to remove support witnesses and 3 stage tumble deburring. Once this was complete various grit polishing wheels were used to bring the part to the desired finish. The parts were then steam cleaned to remove any polishing / finishing residue and then put through nitric acid passivation. However, further details regarding powder bed fusion printing are beyond the scope of this case report.

### Operation

At surgery the right latissimus dorsi muscle was harvested as a pedicled muscle flap based on the thoracodorsal artery with the patient in a left lateral decubitus position. The harvested muscle was placed in the right axilla and the donor wound closed. The patient was placed in a supine position and an elliptical skin and subcutaneous incision was made to excise the biopsy scar. A full thickness skin and subcutaneous flap was raised above and below the incision and the underlying pectoralis major muscle exposed. A wide local excision of the pectoralis major and minor muscles, the tumor deep to the muscle, and the intercostal muscles were then carried out. The second to fourth ribs were divided laterally with a costotome, and the right hemi-sternum and manubrium divided medially with the Hall saw. The 3-D test model in resin was used as a template to determine the cuts. Cutting guides, manufactured in a sterilizeable stereolithographic resin namely, Dental SG and printed on a Form Labs Form 2 printer, were supplied but not deem necessary as the 3-D test model provided a suitable guide for the cuts and to make minor adjustments to the rib and sternal ends. The resected specimen was removed for histology. A 3–5 cm all around macroscopic margins were achieved. An intra-operative frozen section histological examination for microscopically clear margins was omitted to save operative and general anesthetic time [8].

**Fig. 5:**
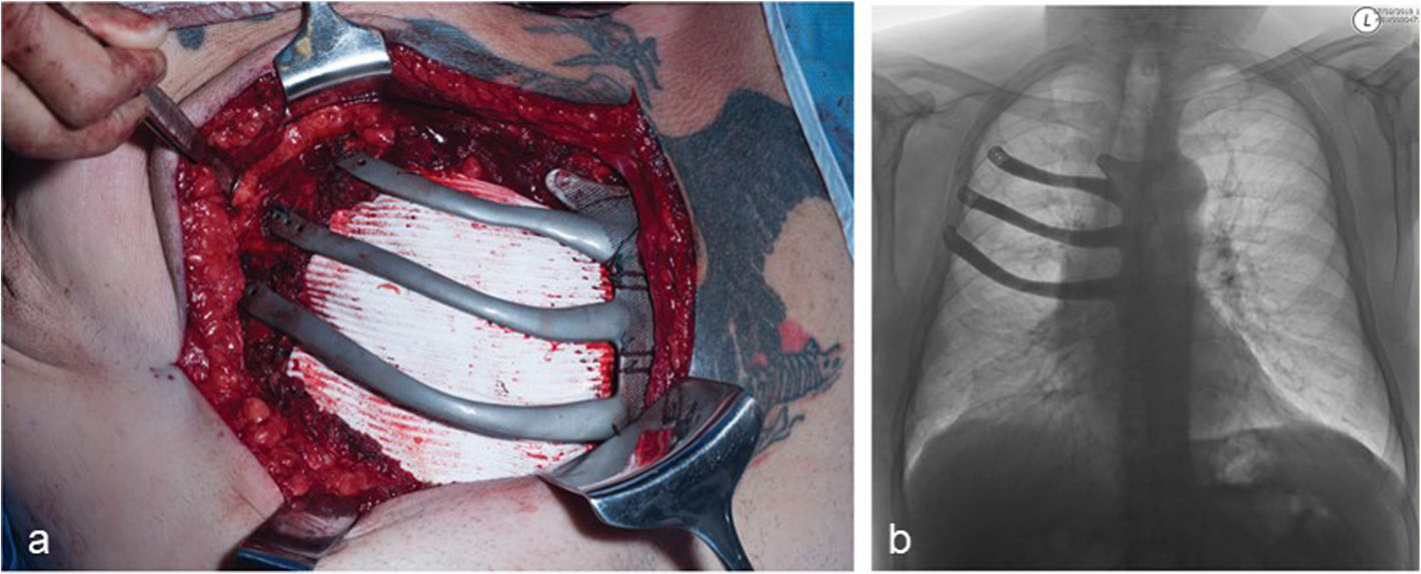
The implant securely in place at surgery (**a**) and on the follow-up chest radiograph (**b**)

To reconstruct the defect the 3-D test model of the implant was first tested on the resected area and was found to slot in and fit the defect perfectly. A Gore-Tex® mesh was secured to the under-surfaces of the ribs and sternum with interrupted ethibond 2/0 sutures to prevent lung herniation through the 3-D implant. The 3-D printed titanium implant, prepared earlier, was then placed on the surfaces of the remaining fragments of the ribs and the sternum and secured into place with ethibond 5 sutures (Fig. 5a), which is a routine practice for securing MMCP to the chest wall. The implant, unlike other titanium devices that are secured with screws, was not secured with screws due to the osteoporotic nature of the ribs and sternum, a narrowed remaining hemi-sternum and predisposition of screws to migrate out of ribs over time [9]. The skeletal reconstruction was then covered with the previously harvested pedicled latissimus dorsi muscle flap and the wound closed in layers.

## Results

Histology confirmed an 80 mm, grade 1 chondrosarcoma. A minimum of 2 cm all round tumor-free microscopic margins on the ribs and sternum were achieved. During the post-operative recovery period regular chest physiotherapy exercises were provided. The hospital discharge was delayed due to prolonged drainage of serous fluid from the harvest site. At 18 month follow-up the patient was asymptomatic and walking without a walking aid and the tremor of his limbs had resolved. He described no breathlessness, pain, discomfort or paradoxical movement at the implant site. On the post-operative chest radiograph and 18-month follow-up CT scan of the thorax, abdomen and pelvis there was no gross evidence of dislocation or tumor recurrence (Fig. 5b).

### Comments

Chondrosarcoma of the chest wall is rare and the natural history and prognosis extremely variable. As chondrosarcoma is largely resistant to conventional chemotherapy and radiotherapy, surgical resection has been the cornerstone of treatment [10]. Following wide local excision the 10 year survival rate is 92% and the local recurrence rate 4% [1]. However, a full thickness wide local excision of the tumor and chest wall leaves a large defect. The reconstruction is complex, challenging, time consuming and requires a combination of skeletal reconstruction and soft tissue cover to minimize a loss in anatomical shape and physiological function of the chest wall.

In the present case, the traditional skeletal reconstruction with a mesh and MMCP was less than ideal as the proposed resection of the right hemi-sternum would have left a large defect and a very narrow left hemi-sternum. The resulting narrowed left hemi-sternum had the potential for stress fracture, and required a robust prosthetics to stabilise the anterior chest wall. The MMCP, traditionally used to reconstruct this defect, would not have met this requirement as it would have been difficult to mould and secure in place. This also increased the potential for dislocation and subsequent paradoxical movement during respiration. Furthermore, due to his underlying coronary artery disease and co-morbidities we were keen to keep the anaesthetic time short. As preparing the MMCP at the time of surgery is time consuming, this was another reason to look for an alternative solution to save time under a general anaesthetic. The estimated operative time saved for this patient was about forty-five minutes by not having to make a prosthesis during the operation.

Advances in 3-D laser printing technology of titanium prostheses have allowed surgical reconstruction of skeletal fractures, particularly in maxillofacial reconstructive surgery [11–16]. The use of this technology was noted to improve the ease of skeletal reconstruction, seemed an ideal solution, was selected for this reconstruction, and as demonstrated met all requirements for a safe and effective alternative. The design of previously used titanium implants for chest wall reconstruction appeared complex or non-anatomical [13–16]. Using individual titanium plates for reconstructing each rib had the potential for movement or displacement, as over time the screws holding the plate to the rib are known to migrate out of the ribs [9]. Securing the implant medially with screws to the remaining narrowed and osteoporotic hemi-sternum also had the potential of causing fracture of the narrowed hemi-sternum, thereby destabilising the chest wall. To avoid these complications, aiming for simplicity and for an anatomical reconstruction to preserve the chest wall shape and physiological function, we designed the present single-piece titanium implant using 3-D digital reconstruction software (Mimics Medical 19, Materialise, Leuven, Belgium, Netherlands and Freeform Plus, Geomagics Inc. USA) from the patients’ own CT scan [4, 5].

Titanium material has a high strength-to-weight ratio, does not flex with movement at joints and can integrate with bone. At our centre maxillo-facial surgeons have used 1.2 mm thick titanium metal plates for load-bearing mandibular reconstruction, with satisfactory long-term results [6, 17]. Ribs are non-weight-bearing long bones and their main function is to maintain the shape of the chest wall. The sternum provides stability anteriorly. With the single-piece ribs and hemi-sternal implant we anticipated minimal movement at the fixation points. Hence, expecting no future risk of fatigue fracture at the fixation points we chose to maintain the 1.2 mm thickness of metal plates for the implant. Long-term follow up is required to establish whether the thickness adopted is adequate.

With the help of our 3-D Maxillofacial Laboratory in Swansea, and Medical Applications Group, Product Design and Research, Cardiff Metropolitan University, the implant was first printed in-house as a 3-D resin prototype and tested, found suitable and then finally printed in titanium alloy by a third party vendor, Renishaw, UK, using a selective laser melting printing process. At surgery it was found to slot in and fit the defect perfectly. The implant was secured into place using the routine standard technique for securing a MMCP with non-absorbable sutures instead of screws, which are used for securing titanium devices. At follow-up on clinical examination and on CT scan of the chest there were no suggestions of dislocation or paradoxical movement. Specifically designing the implant for it to slot into the resected defect, fit onto the ribs and sternum and then firmly securing the implant with interrupted Ethibond sutures to the ribs and sternum has so far prevented dislocation of the implant, confirming this to be a suitable technique. The implant not only helped preserve the anatomical shape and physiological function of the chest wall and thoracic spine but also had a positive psychological impact on the recipient. Tremor of his limbs had resolved and there was a clinically significant improvement in his quality of life.

The planned excision ideally required a 2 cm excision margin. This was identified by creating a 3D model of the tumour from the CT data and exporting this to the Freeform software. The software was digitally instructed to off-set the tumour by 2 cm. The tumour was thus easily inflated by 2 cm in all directions and provided a visual reference of the tumour resection margins in 3D space. No special segmentation techniques were required as the sarcoma margins were easy to threshold-out with clear margins. The clean margins on Fig. 3a may be an artefact of the segmentation process. Manual segmentation was preferred over automated segmentation for visual clarity and satisfaction of adequate resection margins. This approach guided the surgery to achieve clear all round macroscopic and microscopic margins. With wide local excision there is a 4% risk of recurrence [1]. Hence, a long term follow up is required to monitor for recurrence.

The study is limited to a single case experience introducing a novel approach to chest wall reconstruction using emerging technology where an exact replica of the ribs and hemi-sternum to be resected is possible prior to surgery with 3D imaging, whereas only a hand-sculpted approximation is possible with MMCP during surgery. A large controlled series is required to determine whether the titanium model would be superior to MMCP in terms of resistance to fatigue factures or future paradoxical movement, and a study of a larger cohort of patients and a longer term follow-up of 5 years to establish overall survival and disease free survival to fully validate the technique and results. Whilst the technique is a favourable approach to traditional methods of chest wall reconstruction, this rigid single-piece implant, like the MMCP, limits movement at joint articulation sites. Further research for allowing movement at joint sites by innovative designing; using different materials to mimic the sterno-costal joints; and the possibility of using bio-printed chest wall implants is required. Another disadvantage could be a reduced clarity of post-operative CT imaging due to the radio-opaque nature of the implant. However, titanium does not produce large amounts of scatter seen from other alloys such as amalgam and stainless steel. In our experience this has not been an issue [18].

## Conclusion

The use of 3-D laser printing technology to construct a custom-made, anatomical, 3-D printed titanium ribs and hemi-sternum chest wall implant is a useful adjunct to the surgeon that helps reduce the technical challenge and operative time required for reconstructing large chest wall skeletal defects. Our techniques of segmentation, editing, creating the computer aided design for the implant, then using 3-D laser printing to fabricate a custom-made anatomical titanium chest wall ribs and hemi-sternum reconstruction is feasible, safe and provides a satisfactory result. A personalized 3-D printed titanium chest wall implant helps preserve the anatomical shape, structure and function of the thorax. Nevertheless, future studies in a larger cohort of patients and long term follow up is required to validate the clinical efficacy and safety of this technology for chest wall reconstruction.

## Data Availability

All information pertaining to the study, namely pictures, patient consent and operation notes is available for review.

## Abbreviations

3-D: Three-dimensional
CT: Computed tomography
MMCP: Methyl-methacrylate cement prosthesis
STEMI: ST elevation myocardial infarction
STL: Stereolithographic files
WLE: Wide local excision

## References

[1] Björn W, Henrik CF (2009). Surgical treatment is decisive for outcome in chondrosarcoma of the chest wall: a population-based Scandinavian sarcoma group study of 106 patients. J Thorac Cardiovasc Surg.

[2] van Geel AN, Wouters MWJM, Paul TEL, Schmitz M, Verhoef C (2011). Chest Wall resection for adult soft tissue sarcomas and Chondrosarcomas: analysis of prognostic factors. World J Surg.

[3] Khullar OV, Fernandez FG (2017). Prosthetic reconstruction of the Chest Wall. Thorac Surg Clin.

[4] Mitsouras D, Liacouras P, Imanzadeh A, Giannopoulos AA, Cai T, Kumamaru KK (2015). Medical 3D printing for the radiologist. RadioGraphics.

[5] George E, Liacouras P, Rybicki FJ, Mitsouras D (2017). Measuring and establishing the accuracy and reproducibility of 3D printed medical models. RadioGraphics.

[6] Goodson AMC, Evans PL, Goodrum H, Sugar AW, Kittur MA (2017). Custom-made fibular "cradle" plate to optimise bony height, contour of the lower border, and length of the pedicle in reconstruction of the mandible. Br J Oral Maxillofac Surg.

[7] Agapovichev AV, Kokareva VV, Smelov VG, Sotov AV (2016). Selective laser melting of titanium alloy: investigation of mechanical properties and microstructure. IOP Conf Series: Mater Sci Eng.

[8] Anderson ME, Miller PE, van Nostrand K, Vargas SO (2014). Frozen section versus gross examination for bone marrow margin assessment during sarcoma resection. Clin Orthop Relat Res.

[9] Ng CS (2015). Recent and future developments in Chest Wall reconstruction. Semin Thorac Cardiovasc Surg.

[10] Andreou D, Ruppin S, Fehlberg S, Pink D, Werner M, Tunn PU (2011). Survival and prognostic factors in chondrosarcoma: results in 115 patients with long-term follow-up. Acta Orthop.

[11] Eufinger H, Wittkampf AR, Wehmöller M, Zonneveld FW (1998). Single-step fronto-orbital resection and reconstruction with individual resection template and corresponding titanium implant: a new method of computer-aided surgery. J Craniomaxillofac Surg.

[12] Aragón J, Pérez MI (2016). Dynamic 3D printed titanium copy prosthesis: a novel design for large chest wall resection and reconstruction. J Thorac Dis.

[13] Yang H, Tantai J, Zhao H (2015). Clinical experience with titanium mesh in reconstruction of massive chest wall defects following oncological resection. J Thorac Dis.

[14] Dzian A, Živčák J, Penciak R, Hudák R (2018). Implantation of a 3D-printed titanium sternum in a patient with a sternal tumor. World J Surg Oncol.

[15] Aranda JL, Jiménez MF, Rodríguez M,Varela G., Tridimensional titanium-printed custom-made prosthesis for sternocostal reconstruction, Euro J Cardio-Thorac Surg. 2015;48(4):e92–4. 10.1093/ejcts/ezv265. 10.1093/ejcts/ezv265 26242897

[16] Erin Capps S, Shiller M, Cheek S, Oza U, Konduri K (2009). Chest wall chondrosarcoma. Proc Bayl Univ Med Cent.

[17] Pacifici L, DE Angelis F, Orefici A, Cielo A (2017). Metals used in maxillofacial surgery. Oral Implantol (Rome).

[18] Billè A, Okiror L, Karenovics W, Routledge T (2012). Experience with titanium devices for rib fixation and coverage of chest wall defects. Interact Cardiovasc Thorac Surg.

